# Effect of optical impression, timing of dentin sealing application and cavity design optimization on microleakage of ceramic onlays: an in vitro study

**DOI:** 10.1186/s12903-026-08348-w

**Published:** 2026-04-25

**Authors:** Hanan Ahmed Nabil Soliman, Ahmed Ismail Taha

**Affiliations:** 1 Conservative Department, Faculty of Dentistry, Kafr Elsheikh University, Kafr Elsheikh, Egypt; 2 Prosthodontic Department, Faculty of Dentistry, Kafr Elsheikh University, Mubark Road, 33511 Kafr Abu Tabl, Kafr Al Sheikh Governorate, 6860404 Egypt

**Keywords:** Digital impression, Immediate dentin sealing, Onlay, Microleakage, Dye penetration

## Abstract

**Statement of problem:**

Immediate dentin sealing (IDS) is used to improve bonding of indirect restoration. However, interaction between polyether impressions may affect the future bonding of indirect restoration. So, digital impressions with IDS may have a better impact on microleakage.

**Purpose:**

The current in vitro study aimed to investigate the effect of using digital impression versus polyether impression and different dentin sealing techniques on the microleakage of lithium disilicate onlays.

**Material and methods:**

A total of 60 identically sized human mandibular molars were allocated into two groups (*n* = 30): group DI, which uses a digital impression, and group CI, which uses a polyether impression. After preparation of all teeth for onlay, based on the dentin sealing technique, these groups were further divided into three subgroups (*n* = 10): subgroups I, II, and III. Dentin sealing was postponed in subgroup I prior to cementation. Immediate dentin sealing was applied prior to impression processes in subgroups II and III, plus cavity design optimization (CDO) for subgroup III. All teeth received e.max CAD onlay restoration (Ivoclar Vivadent, Schaan, Liechtenstein). The specimens were exposed to thermocycling with bath temperatures of 5 °C and 55 °C for a total of 5000 cycles. The specimens were then submerged in 0.5% aqueous Rhodamine B dye for 48 h. Buccolingual sections were examined under confocal microscopy. Microleakage was scored (0–3). Data were analyzed using the Scheirer–Ray–Hare test (α = 0.05).

**Results:**

Microleakage was significantly lower in DI than CI (*P*=.0046). Delayed dentin sealing (subgroup I) showed significantly higher microleakage than IDS (subgroup II) and IDS + CDO (subgroup III) (*P*<.001), with no significant difference between IDS and IDS + CDO.

**Conclusions:**

Digital impressions were associated with significantly lower microleakage compared to polyether impressions. IDS, with or without CDO, significantly reduced microleakage compared to delayed sealing.

**Clinical implications:**

Combining digital impressions with IDS may enhance the marginal seal and longevity of ceramic onlays.

## Introduction

Bonded indirect restorations, such as onlays, are conservative and esthetic solutions for compromised posterior teeth, supported by advances in adhesive dentistry and material science [1, 2]. Their clinical success depends on precise marginal adaptation and an effective seal against microleakage [3].

A critical factor for achieving this seal is the management of exposed dentin. Traditional delayed dentin sealing (DDS), where adhesive is applied just before final cementation, presents risks of postoperative sensitivity, bacterial contamination under provisional restorations, and compromised restoration seating [4]. Immediate dentin sealing (IDS)—applying adhesive to freshly cut dentin after preparation and before impression—has been shown to improve bond strength, reduce sensitivity, and enhance marginal adaptation [5, 6].

However, a potential drawback of IDS is its interaction with polyether impression materials, as the oxygen-inhibited layer (OIL) on the cured adhesive can affect impression accuracy [7, 8]. To mitigate this, applying a thin, cured layer of flowable resin composite over the sealed dentin—termed resin coating or cavity design optimization (CDO)—has been proposed [9, 10]. This layer also optimizes cavity geometry by reducing the configuration factor (C-factor), potentially lowering polymerization stress at the margins [10].

Concurrently, digital impression techniques have become widespread, eliminating material-related interactions and potentially offering superior accuracy [11, 12]. While both IDS and digital workflows are individually advocated, comparative data on their combined effect on a critical outcome such as microleakage are lacking. Most existing studies have evaluated these factors in isolation—either comparing impression techniques or dentin sealing protocols—but not in an integrated factorial design that reflects contemporary clinical workflows.

Therefore, this study aimed to fill this gap by simultaneously investigating the effects of impression technique and dentin sealing protocol on the microleakage of lithium disilicate onlays. Specifically, the objectives were to (1) compare microleakage of lithium disilicate onlays fabricated using a direct digital scanning workflow versus an indirect digitization workflow using polyether impressions and stone casts and (2) evaluate the effect of DDS, IDS, and IDS combined with cavity design optimization (IDS + CDO) on microleakage. The corresponding null hypotheses were: firstly, there is no difference in microleakage between onlays fabricated from direct digital scans and those from indirect digitization of polyether impressions. Secondly, there is no difference in microleakage among onlays fabricated using DDS, IDS, and IDS + RC protocols.

## Materials and methods

### Sample size calculation

The Kafr Elsheikh University Ethics Committee approved the study protocol (No. KFSIRP200-158). Patients were asked to sign an informed consent form before their teeth were extracted.

The sample size was calculated by G*Power [13] using “F tests: ANOVA: Fixed effects, special, main effects, and interactions,” and the type of power analysis is “a priori.” Compute the required sample size given α, power, and effect size. When the effect size is f = 0.5 (large, per Cohen’s conventions for ANOVA), which is based on previous studies [14–16], α = 0.05, power = 0.95, and the number of groups = 6, this typically yields 55 total samples for main effects. Thus, the sample size was adjusted upward for 60 molars: 10 samples per subgroup.

### Specimen preparation and grouping

**Fig. 1:**
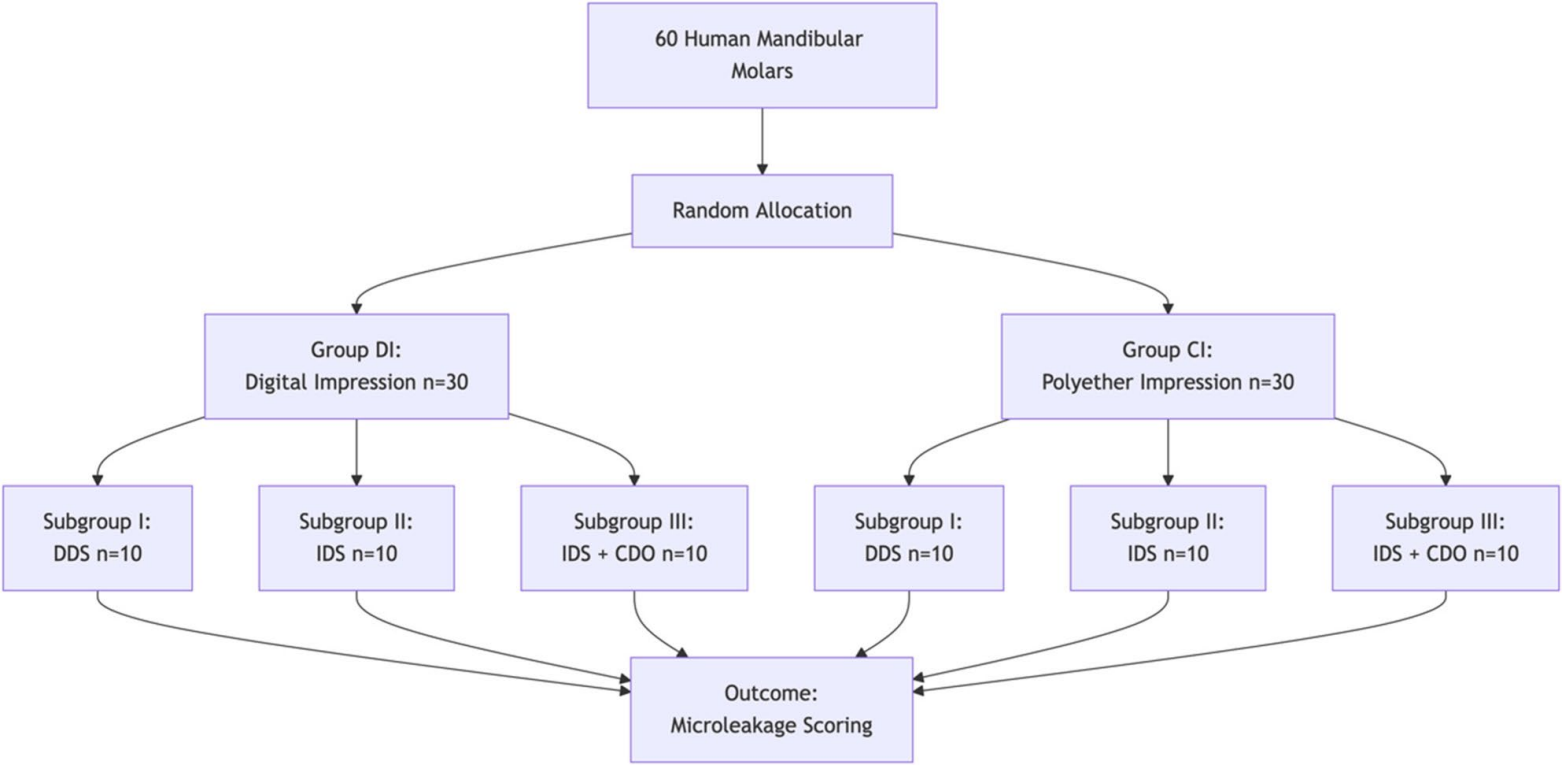
Study design flowchart illustrating the allocation of specimens into groups based on impression technique and dentin-sealing protocol

A total of 60 identically sized human mandibular molars were extracted for orthodontic or periodontal purposes; the buccopalatally and mesiodistally dimensions of each tooth were measured using a digital caliper. A 0.5 mm discrepancy was deemed acceptable for each measurement in order to standardize tooth dimensions; the teeth were examined under ×10 magnification to confirm the absence of caries, restorations, or anatomical defects. The collected teeth were cleaned of calculus and soft tissue residues with a hand scaler and pumice prophylactics and then preserved in distilled water at 37 °C until use. Teeth were randomly assigned to six subgroups (*n* = 10), as shown in Fig. 1.

For standardization, an experienced operator (A. T.) performed all preparations utilizing a high-speed handpiece with a cooling system in accordance with established recommendations and a cylindrical diamond bur with rounded tip 446KR.011 (Jota 1925, Ituren, Switzerland). Two researchers used a periodontal probe (North Carolina, Hu-Friedy^®^, USA) to confirm the dimensions.

All teeth were prepared with a mesio-occlusal-distal onlay design with the following dimensions: Occlusal depth was 1.5 mm, isthmus depth was 3 mm, and width was 2 mm. Proximal boxes were prepared to a depth of 1 mm above the cementoenamel junction. The buccal and lingual cusps were reduced by 2 mm to complete the onlay preparation with buccal ledge preparation of 1 mm thickness [17].

Following preparation, all molars were allocated into two groups (*n* = 30) at random: group DI, which uses a digital impression approach, and group CI, which uses a traditional impression technique (polyether impression). Based on the dentin sealing technique, these subjects were further divided into three subgroups (*n* = 10): subgroups I, II, and III.

### Immediate dentin sealing protocol

For subgroups II and III, IDS was performed immediately after preparation. First, the dentin surface was cleaned with phosphoric acid etchant (37%) (Kerr, Orange, CA, USA) for 1–3 s, rinsed thoroughly, and gently dried. A primer (FL-Bond II, Shofu, Kyoto, Japan) was applied to dentin for 20 s and gently dried. Adhesive (FL-Bond II, Shofu, Kyoto, Japan) was then applied for 15 s and light-cured for 20 s (≥ 1000 mW/cm², Bluephase, Ivoclar Vivadent). For subgroup III (IDS + CDO), a thin layer of bulk-fill flowable composite (Smart Dentine Replacement (SDR), Caulk, Dentsply/Sirona, USA) was chosen for its claimed low polymerization stress and bulk-fill capability, applied, and light-cured for 40 s after applying glycerin gel (Oxyguard, Kuraray Noritake, Tokyo, Japan). Excess material was removed with diamond burs, and surfaces were polished. For Group CI subgroups II and III, prior to polyether impression, the sealed surface was cleaned with a pumice slurry and rinsed to remove the oxygen-inhibited layer. All steps followed manufacturers’ instructions.

### Impression and fabrication

**Fig. 2:**
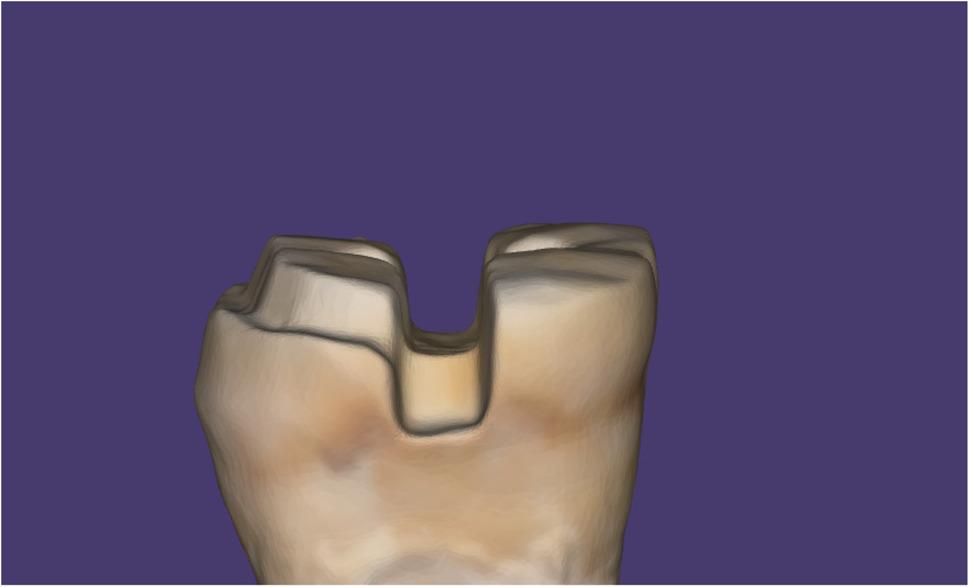
Intraoral scan of an onlay preparation acquired directly from the tooth (digital impression group)

Group DI: Digital impressions were taken directly from the tooth using an intraoral scanner (Medit i700, MEDIT Corp., Seoul, Republic of Korea), as seen in Fig. 2.

**Fig. 3:**
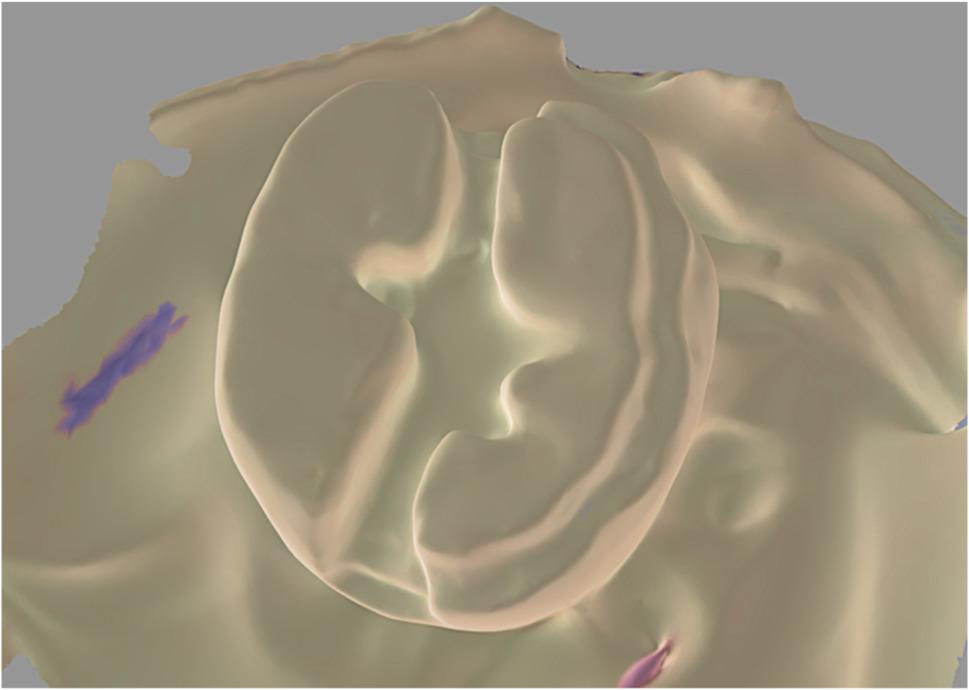
scan of a stone die poured from a polyether impression (conventional impression group)

Group CI: Polyether impressions (Impregum, 3 M ESPE, Ontario, Canada) were poured in extra-hard stone, and the stone dies were scanned extraorally with the same scanner as shown in Fig. 3.

STL files were imported into CAD software (DentalCAD 3.0 Galway 2021, Exocad, Darmstadt, Germany) for designing onlays with a uniform 60 μm cement spacer. Onlays were milled from e.max CAD blocks (Coritec 250i, imesicore GmbH, Eiterfeld, Germany) and crystallized (Vita Vacumat 6000 M; VITA Zahnfabrik GmbH, Bad Säckingen, Germany).

### Provisionalization

Provisional restorations were fabricated directly on the tooth preparation using bis-acryl resin (Protemp 4, 3 M ESPE, Seefeld, Germany) and cemented with non-eugenol temporary cement (Temp-Bond NE, Kerr, Orange, USA). The provisional restorations remained in place for one week to simulate a typical clinical provisionalization period before being removed for final cementation.

### Cementation

After provisional removal, sealed surfaces were cleaned with airborne particle abrasion (Cojet, 3 M ESPE, Seefeld, Germany). Onlays were etched with 4.5% hydrofluoric acid gel (Porcelain etch, Ultradent Products, Cologne, Germany) for 20 s, rinsed, dried, and silanized (Silane, Ultradent Products, Cologne, Germany) for 60 s. A dual-cure resin cement (RelyX Ultimate, 3 M ESPE) was selected for its compatibility with the adhesive and common clinical use [18]. It was applied to the restoration, which was seated under firm finger pressure for 1 min, ensuring complete seating and adaptation before light-curing. Excess cement was removed, and margins were light-cured for 20 s per surface under glycerin gel to prevent oxygen inhibition. All materials were used per manufacturer instructions.

### Aging

After 48 h of water storage, specimens were exposed to thermocycling with bath temperatures of 5 °C and 55 °C for a total of 5000 cycles. The bath time for each temperature was 20 s, and the time of transfer between baths was 2 s.

### Microleakage evaluation

Root apices were sealed with nail varnish, leaving a 1 mm margin around the restoration uncovered [19]. Specimens were immersed in 0.5% Rhodamine B dye for 48 h [20], rinsed, and sectioned buccolingually into four slices using an Isomet saw (Buehler, Lake Bluff, IL, USA) under water cooling [21]. The two outermost slices were discarded. Sectioning may cause minor apparent dimensional differences. The three middle sections per tooth were polished and examined under confocal laser scanning microscopy [22] at ×10 magnification (LSM 510 Meta Confocal Microscope, Zeiss, Germany), and images were recorded.

**Fig. 4:**
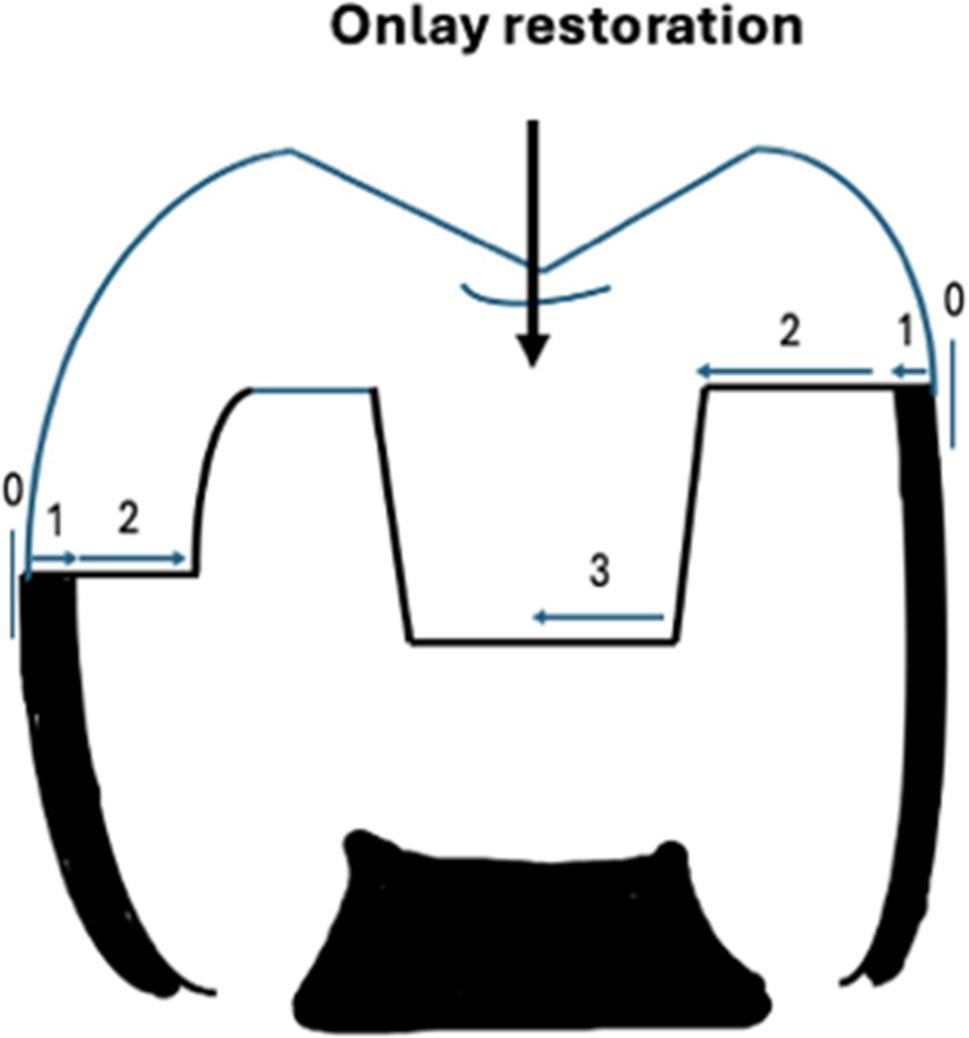
Schematic representation of the 0–3 scoring scale for dye penetration at the restoration margins: (1 = moderate dye penetration to the enamel, 2 = dye penetration at the dentin level without including the pulpal floor of the cavity, and 3 = dye penetration up to the pulpal floor)

**Fig. 5:**
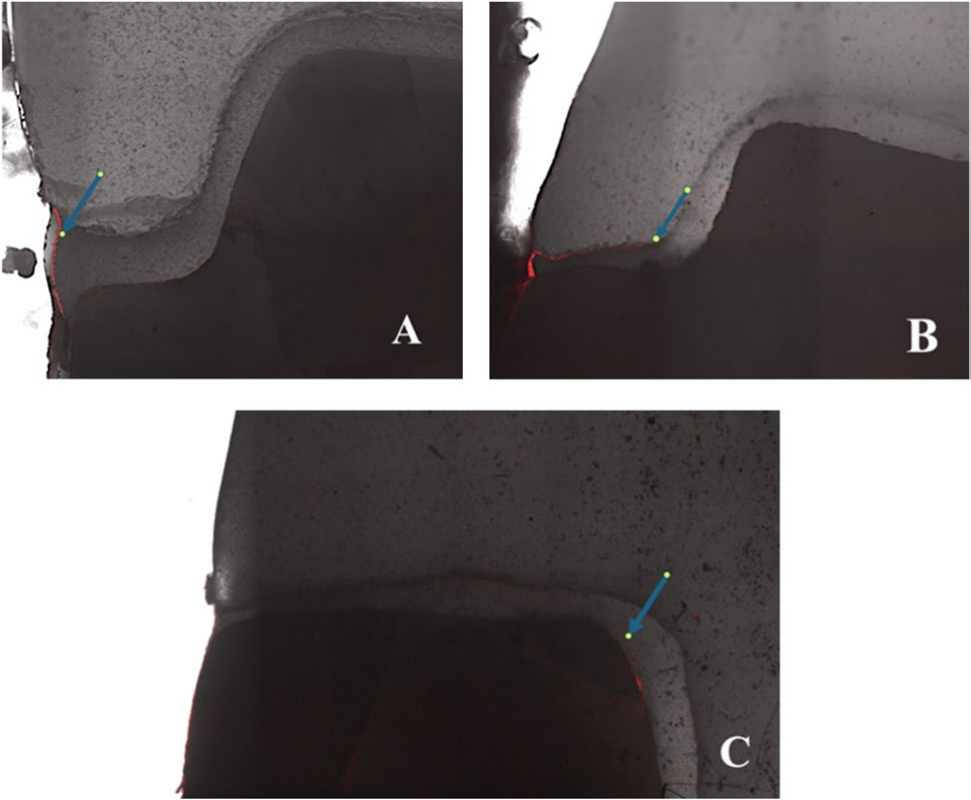
**A-C**, Confocal laser scanning micrographs (×10) illustrating representative microleakage scores: **A** score 0 (no penetration), **B** score 1 (penetration limited to enamel), **C** score 2 (penetration at the dentin level without including the pulpal floor of the cavity)

Dye penetration was scored by two calibrated examiners blinded to group allocation (inter-examiner kappa = 0.88) according to the scale (0–3) [3] depicted in Fig. 4, with representative micrographs shown in Figs. 5A-C. The scores from the three sections per tooth were averaged to yield a single microleakage score per tooth for statistical analysis.

### Statistical analysis

Data were entered and analyzed using IBM-SPSS software (IBM Corp. released 2020, IBM SPSS Statistics for Windows, Version 27.0. Armonk, NY: IBM Corp.). The averaged, ordinal microleakage scores were not normally distributed. Therefore, a non-parametric two-way analysis was performed using the Scheirer-Ray-Hare test, the factorial extension of the Kruskal-Wallis test, with α = 0.05. This test is appropriate for analyzing ranked data from a factorial design when the assumptions of parametric ANOVA are not met [23]. Pairwise comparisons were performed with Bonferroni correction.

## Results

The frequency distributions of microleakage scores are presented in Table 1. Score 1 was the most frequent in both main groups, while score 2 showed the lowest frequency in group DI, and score 0 showed the lowest frequency in group CI.

**Table 1:** The frequencies of microleakage score

Category	Microleakage score			
	0	1	2	3
Group				
DI	9 (30%)	19 (63.3%)	2 (6.7%)	0 (0%)
CI	3 (10%)	17 (56.7%)	10 (33.3%)	0 (0%)
Subgroup				
I	2 (10%)	6 (30%)	12 (60%)	0 (0%)
II	4 (20%)	16 (80%)	0 (0%)	0 (0%)
III	6 (30%)	14 (70%)	0 (0%)	0 (0%)

Data is *N* (%). refers to the number and percentage of teeth within each group or subgroup exhibiting the given microleakage score

Mean ranks of microleakage scores are presented in Table 2. The highest mean rank (greatest microleakage) occurred in the delayed-sealing subgroup (I) of the conventional impression group, while the lowest mean rank was observed in the IDS + CDO subgroup of the digital impression group.

**Table 2:** The mean ranks of microleakage score

Group	Subgroup			
	I	II	III	Total
DI	1	0.7	0.6	0.766667
CI	2	0.9	0.8	1.233333
Total	1.5	0.8	0.7	

Data is presented as mean ranks

As shown in Table 3, the two-way Scheirer-Ray-Hare test revealed a statistically significant main effect for the impression workflow factor (H = 8.03, *P*=.0046), with the DI group showing a lower mean rank (0.77) than the CI group (1.23), indicating significantly less microleakage with direct digital scanning.

**Table 3:** Two-way ANOVA on ranked data (Scheirer-Ray-Hare Test) for microleakage score

Test	SS	df	H	*p*-value	Sig.
Impression techniques (DI vs. CI)	3.266667	1	8.030556	0.004599	yes
Sealing protocol (DDS vs. IDS vs. IDS + CDO)	7.6	2	18.68333	0.000088	yes
Inter (Impression × Sealing interaction)	2.133333	2	5.244444	0.072641	no
Within	11	54			
Total	24	59			

Mean ranks for main effects and subgroups are presented in Table 2 and the Results section. The Scheirer–Ray–Hare test was used due to the non-normal distribution of ordinal microleakage scores

*df  *degrees of freedom, *Sig. *significance,* SS *Sum of squares

**Fig. 6:**
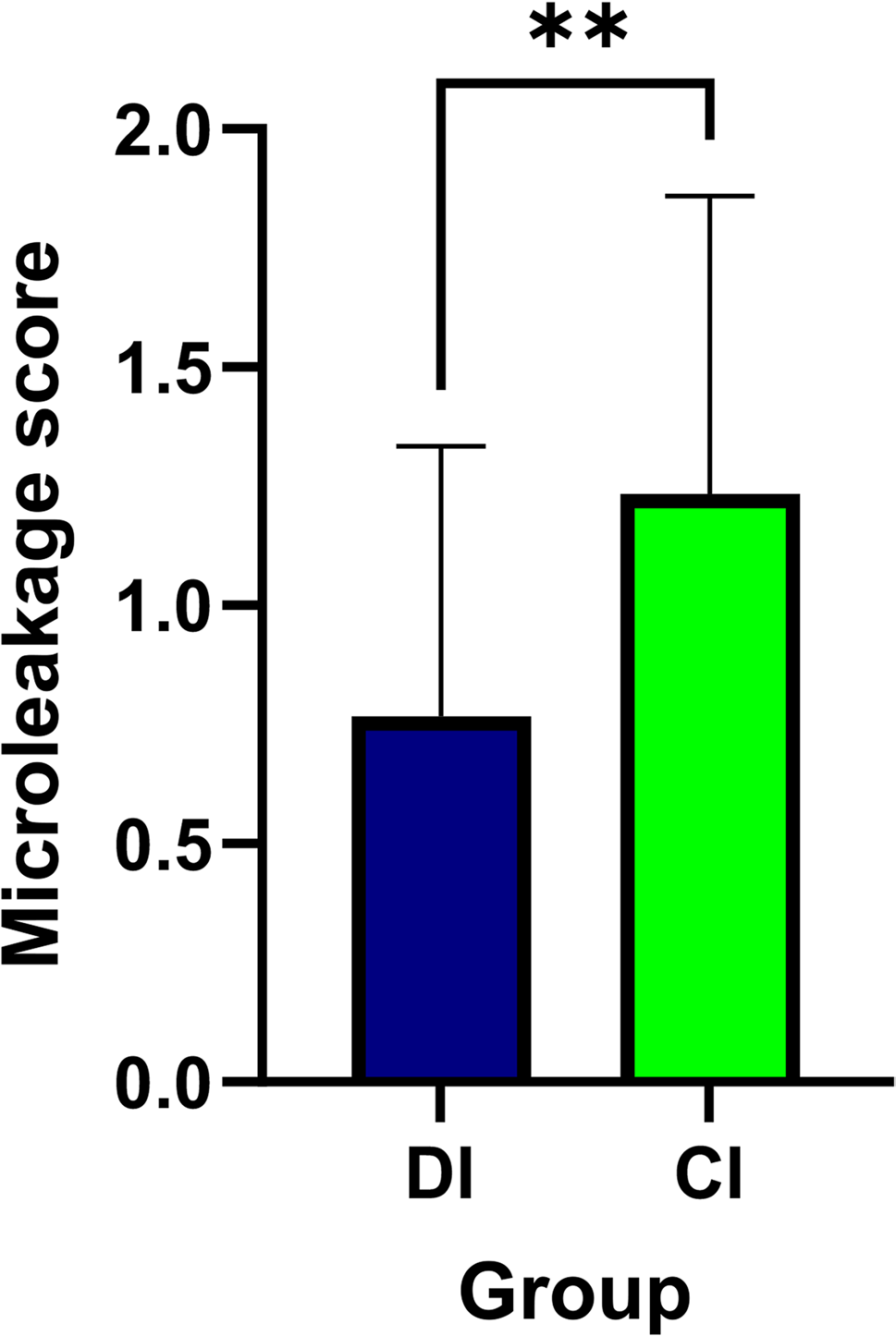
Box-plot comparing microleakage scores between digital impression (DI) and conventional impression (CI) groups. Asterisks indicate statistically significant differences between groups (**P < .01)

**Fig. 7:**
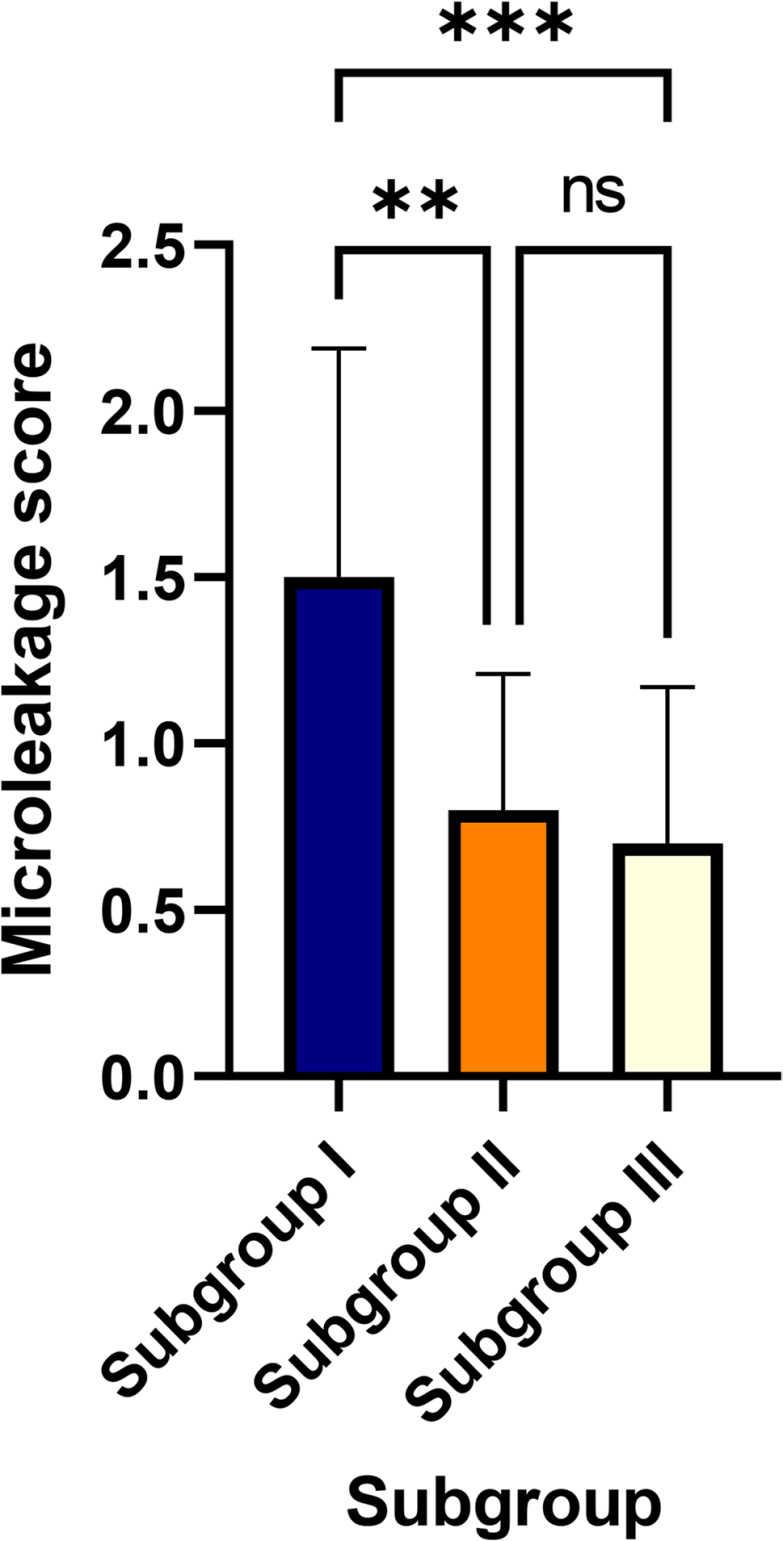
Box-plot comparing microleakage scores among the three dentin-sealing subgroups (I: DDS, II: IDS, III: IDS + CDO). Asterisks indicate statistically significant pairwise differences (**P < .01, ***P < .001)

A statistically significant main effect was also found for the dentin sealing protocol (H = 18.68, *P*<.001). Pairwise comparisons showed that the DDS subgroup (mean rank = 1.5) had significantly higher microleakage than both the IDS (mean rank = 0.8) and IDS + CDO (mean rank = 0.7) subgroups. There was no significant difference between the IDS and IDS + CDO subgroups. The interaction effect was not significant (*P*=.0726). The pairwise comparisons between groups are illustrated in Figs. 6 and 7.

## Discussion

This study investigated the combined effects of impression workflow and dentin sealing protocol on the marginal microleakage of lithium disilicate onlays. The results led to the rejection of the two null hypotheses, as a statistically significant difference was found between direct digital scans and indirect digitization of polyether impressions, with digital impressions yielding restorations that exhibited less microleakage. Furthermore, a significant difference was observed among the dentin sealing protocols, with immediate dentin sealing (both with and without an additional flowable liner) resulting in significantly less microleakage compared to the traditional delayed sealing approach.

Digital impressions were associated with significantly lower microleakage than polyether impressions (*P* = .0046). This may be attributed to the elimination of material deformation, stone expansion, and additional handling errors inherent in the conventional workflow involving stone cast digitization [24, 25]. This finding aligns with the principle that a more accurate initial impression leads to a restoration with superior marginal fit, which is a primary determinant in preventing microleakage. Research by Alshammari et al. [26] directly compared microleakage in crowns fabricated from digital versus polyether impressions and found significantly lower dye penetration in the digital group, attributing this to the superior marginal and internal adaptation achieved through the digital workflow. However, it should be noted that the CI group represented an indirect digitization workflow, not a purely conventional one, which is a study limitation.

Our results are supported by clinical and in vitro studies comparing marginal fit; Abdel-Azim et al. [27] found that lithium disilicate crowns from digital impressions had comparable or better marginal fit than those from conventional impressions. Similarly, a systematic review by Tabesh et al. [25] concluded that for zirconia restorations, digital impressions yielded slightly superior marginal adaptation compared to conventional elastomeric impressions. The present study extends these findings by demonstrating that this advantage in precision translates to a measurable improvement in the clinical outcome of marginal seal, as quantified by reduced microleakage.

The second major finding was that IDS significantly reduced microleakage compared to DDS (*P*<.001), consistent with prior studies [5, 28]. The improved seal likely results from superior adhesive penetration into fresh dentin and reduced contamination risk during provisionalization [29]. Furthermore, the application of adhesive on freshly cut dentin with open tubules facilitates the formation of a thicker, more homogeneous hybrid layer, which serves as a more effective barrier against fluid penetration. A confocal microscopic study by Kasraei et al. [30] demonstrated that IDS resulted in significantly longer resin tags and a more continuous hybrid layer compared to DDS, which correlated with reduced nanoleakage at the dentin interface. In addition, the IDS layer can act as a stress-absorbing intermediary, better accommodating and distributing the polymerization shrinkage stresses generated by the luting cement, thereby protecting the more critical dentin-adhesive interface [31]. Magne et al. [29] provided foundational support for this concept, demonstrating that IDS significantly improved the bond strength of indirect restorations compared to DDS. Our microleakage results provide functional corroboration of this improved interfacial integrity.

Interestingly, the addition of a CDO over IDS did not yield a further significant reduction in microleakage compared to IDS alone under these experimental conditions. This contrasts with some studies advocating CDO as a stress-absorbing layer [28, 32], a discrepancy that may relate to differences in materials, the thickness of the applied layers, or cavity design. It is possible that in a well-controlled in vitro setting with a standardized, non-undercut preparation, the primary benefit of the CDO—optimizing cavity geometry and reducing the configuration (C)-factor—was less pronounced. However, in more complex clinical scenarios with high C-factor cavities, the stress-relieving properties of a flowable resin coating might become more critical for marginal integrity, as suggested by the work of Soares et al. on polymerization stress [31].

This in vitro study has several limitations. The standardized preparation may not reflect the extensive tooth loss often seen clinically. The dye penetration method, while common, is qualitative and subject to interpretation, though we employed calibrated examiners. The absence of mechanical loading limits simulation of oral conditions. The use of adhesive and flowable resin from different manufacturers, though clinically common, may introduce variables in compatibility. Finally, as noted, the comparison was between a direct digital workflow and an indirect digitization workflow via stone casts, not a purely conventional clinical protocol. Future research should incorporate mechanical loading, evaluate performance in more challenging cavity configurations, and consider using the adhesive and flowable of the same brand for better results. Ultimately, randomized controlled clinical trials are needed to validate these findings.

## Conclusion

Within the limitations of this in vitro study:

1. A direct digital impression workflow was associated with significantly lower microleakage than an indirect digitization workflow using polyether impressions and stone casts.
2. Immediate dentin sealing, with or without CDO, significantly reduced microleakage compared to delayed dentin sealing.

These findings support the combined use of digital impressions and IDS to improve the marginal seal of ceramic onlays.

## Data Availability

No datasets were generated or analysed during the current study.
